# Lipid and Glycolipid Isomer Analyses Using Ultra-High Resolution Ion Mobility Spectrometry Separations

**DOI:** 10.3390/ijms18010183

**Published:** 2017-01-18

**Authors:** Roza Wojcik, Ian K. Webb, Liulin Deng, Sandilya V. B. Garimella, Spencer A. Prost, Yehia M. Ibrahim, Erin S. Baker, Richard D. Smith

**Affiliations:** 1 Biological Sciences Division, Pacific Northwest National Laboratory, Richland, WA 99352, USA; roza.wojcik@pnnl.gov (R.W.); ian.webb@pnnl.gov (I.K.W.); liulin.deng@pnnl.gov (L.D.); sandilya.garimella@pnnl.gov (S.V.B.G.); yehia.ibrahim@pnnl.gov (Y.M.I.); 2 Environmental Molecular Sciences Laboratory, Pacific Northwest National Laboratory, Richland, WA 99352, USA; spencer.prost@pnnl.gov

**Keywords:** lipids, glycolipids, isomers, ion mobility spectrometry

## Abstract

Understanding the biological roles and mechanisms of lipids and glycolipids is challenging due to the vast number of possible isomers that may exist. Mass spectrometry (MS) measurements are currently the dominant approach for studying and providing detailed information on lipid and glycolipid presence and changes. However, difficulties in distinguishing the many structural isomers, due to the distinct lipid acyl chain positions, double bond locations or specific glycan types, inhibit the delineation and assignment of their biological roles. Here we utilized ultra-high resolution ion mobility spectrometry (IMS) separations by applying traveling waves in a serpentine multi-pass Structures for Lossless Ion Manipulations (SLIM) platform to enhance the separation of selected lipid and glycolipid isomers. The multi-pass arrangement allowed the investigation of paths ranging from ~16 m (one pass) to ~60 m (four passes) for the distinction of lipids and glycolipids with extremely small structural differences. These ultra-high resolution SLIM IMS-MS analyses provide a foundation for exploring and better understanding isomer-specific biological activities and disease processes.

## 1. Introduction

**Figure 1:**
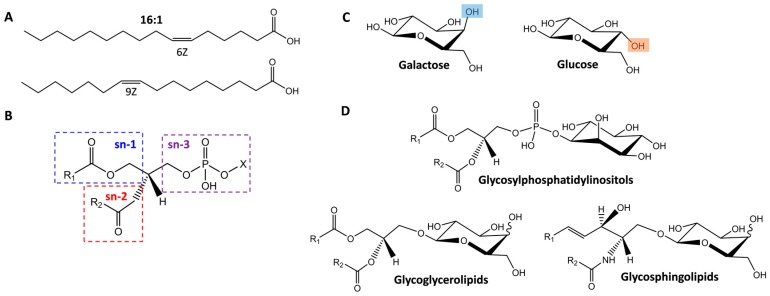
The isomeric differences in lipids and glycolipids. (**A**) Illustrates potential double bond positions in a 16:1 fatty acid; (**B**) Shows possible fatty acid linkage connectivity sites (sn-3 is shown for a phospholipid where X changes depending on the glycerophospholipid type); (**C**) Elucidates the hydroxyl group differences between galactose and glucose which are highlighted in blue and orange and (**D**) Illustrates the three main categories of glycolipids.

Evaluating the role of lipids in diverse biological systems has important implications in understanding disease initiation and progression [1]. Nonetheless, these studies have proven to be quite difficult due to the complexity of lipid structures [2,3]. Lipids are composed of hydrocarbon backbones with fatty acid chains arranged at distinct positions (i.e., sn-1 versus sn-2) with intra-structural position and orientation differences in the double bonds and functional groups such as *cis* and *trans* orientations and distinct double bond locations (Figure 1A,B). Glycolipids have even more isomeric variation as they combine the many different lipid isomers with glycan isomer subunits that have distinct identities, linkage types and connectivities (i.e., linear versus branching) (Figure 1C). Glycolipids are categorized into three main groups, glycosylphosphatidylinositols, glycoglycerolipids, and glycosphingolipids (GSLs), depending on the lipid type present (Figure 1D). In mammalian systems, GSLs either have glucose or galactose attached to the β-linkage at the 1-hydroxyl position on the ceramide (Cer) or sphingoid base, eliciting distinct functions. Galactosylceramides (GalCer) can be sulfated to produce acidic GSLs or sulfatides. However, glucosylceramides (GlcCer) function differently as the addition of galactose to GlcCer can result in lactosylceramide (LacCer), which is the branching point for the globo-, isoglobo-, lacto-, neolacto-, and ganglio-series [4]. In particular, integral membrane glycosyltransferases can convert LacCer to both the neutral and acidic members of the ganglio-series by the sequential addition of different monosaccharide units [5], resulting in glycolipids of potential importance. Specifically, gangliosides are mono- or multi-sialylated GSLs from the ganglio-series, which are the major components of nerve cells (representing more than 10% of the total lipid content) and thought to be possible therapeutics or therapeutic targets for neurodegenerative disorders [6,7,8,9,10]. However, considering all the possible isomers of gangliosides, their specific functions have remained largely unknown due to challenges with current separation techniques. Thus, the ability to distinguish and identify glycolipid isomers is essential for investigating their physiological role and functions in nature and diseases.

Comprehensive lipidomic analyses require a combination of techniques for their identification, separation and quantitation. MS is an essential tool for lipid structural characterization, alongside other techniques, such as X-ray diffraction [11], nuclear magnetic resonance spectroscopy (NMR) [11] and ELISA (enzyme-linked immunosorbent assay) [12]. Currently, most lipidomic and glycolipidomic separations are performed with liquid chromatography (LC) coupled to mass spectrometry (MS) and tandem MS/MS analyses. While normal phase LC [13,14,15], reverse phase LC [2,16,17], silver ion chromatography [18,19] and gas chromatography [20,21] have the utility for separations of some isomers, they have long analysis times and are not universal for all lipid classes [22]. Significant advances in lipid isomer structural elucidation have been made in conjunction with techniques such as ozone-induced dissociation, charge switch derivatization, radical-directed dissociation, collisional-induced dissociation (CID) and photodissociation [23,24,25,26,27]; however, further advances are still needed. Ion mobility spectrometry (IMS) is an appealing technique for lipidomic and glycolipidomic analyses and has previously demonstrated utility for the separation of lipid isomers from distinct categories and subclasses [28,29,30,31,32,33,34]. However, due to the current IMS resolving power limitations, lipid isomers cannot be fully resolved by IMS alone in complex mixtures extracted from biofluids or environmental samples [2].

**Figure 2:**
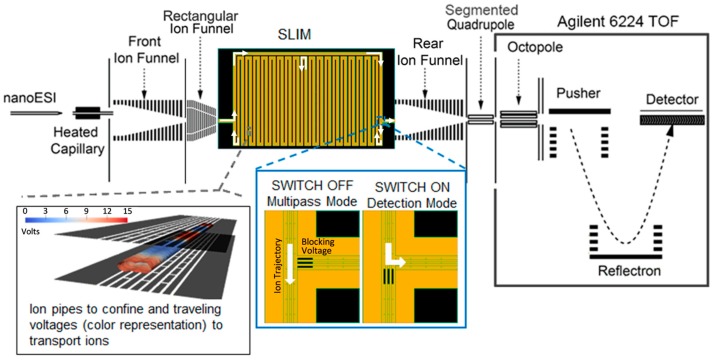
A schematic of the multi-path Structures for Lossless Ion Manipulations (SLIM) ion mobility spectrometry-mass spectrometry (IMS-MS) platform illustrating each component from the source to the mass spectrometer (**top**) with insets showing ion confinement between the two parallel SLIM surfaces (**bottom-left**) and blocking voltage positions for the two switches (**bottom-middle**).

Structures for Lossless Ion Manipulations (SLIM) technology using travelling wave ion mobility spectrometry (TWIMS) [35,36] has recently been shown to enable greatly extended path lengths and provide ultra-high resolution separations. TWIMS relies on using an ion guide with a series of orthogonally arranged electrodes and adjacent electrodes with the opposite phase of radio frequency (RF) voltage. Superimposed time-varying potentials propagate along the ion guide and create a travelling wave that drives ion motion. Ions with mean travelling velocities lower than the travelling wave speed roll over the wave with frequencies dependent on their mobilities [37]. While present commercial TWIMS instruments use stacked ring ion guides [38], SLIM TWIMS uses fields created by potentials applied to arrays of electrodes on two parallel surfaces to generate ion paths. Recently, we introduced both SLIM serpentine ion path as well as multi-pass designs, which enabled extended IMS path lengths and much higher resolving power than previous platforms [35,36,39,40,41,42] (Figure 2). An early single-pass version of this platform was able to separate isomeric *cis* and *trans* positional lipid isomers of glycerophosphatidylethanolamine (PE) and branched oligosaccharides, showing greatly enhanced resolution when compared to conventional IMS drift cell platforms [39]. Herein, we utilized the multi-pass SLIM IMS-MS platform to demonstrate ultra-high resolution IMS separations capable of distinguishing glycerophospholipid and glycolipid isomers with very similar structures.

## 2. Results and Discussion

In this manuscript, we evaluated lipid and glycolipid isomers with ultra-high resolution multi-pass IMS separations. Lipid isomer pairs varied in the double bond orientation (*cis* versus *trans*) and locations on the fatty acid chains. Glycolipids with galactosyl and glucosyl variations, distinct connectivities, and different branching of their glycan subunits were also analyzed. Each isomer pair is discussed in detail below.

### 2.1. Isomer Separation of Double Bond Positions and Cis/Trans Orientations

**Figure 3:**
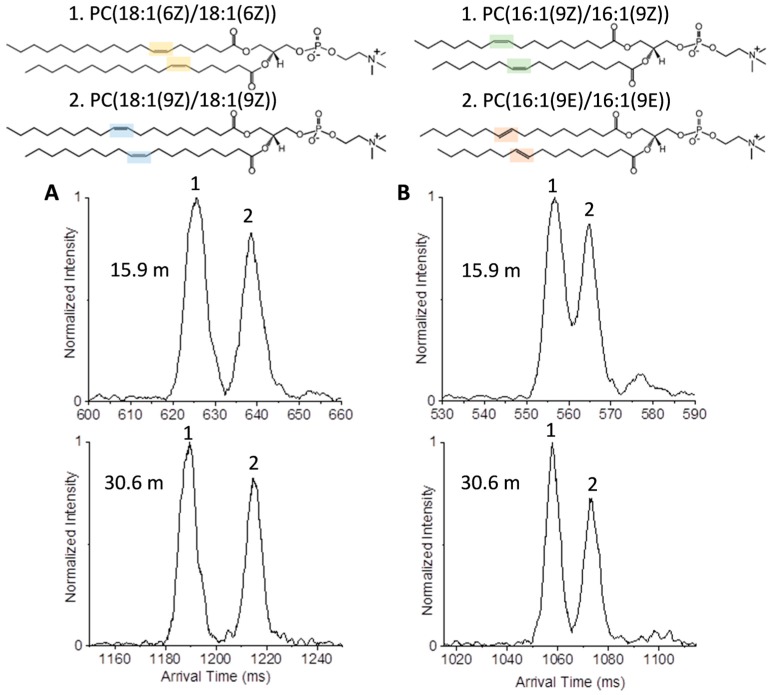
Ultra-high resolution IMS separations of protonated PC lipids with (**A**) distinct double bond positions and (**B**) orientations. (**A**) To study different double bond positions, a mixture of (glycerophosphatidylcholines) PC(18:1(6Z):18:1(6Z)) and PC(18:1(9Z):18:1(9Z)) (*m*/*z* 786.601 for the protonated species) was studied with one (15.9 m) and two passes (30.6 m) in the SLIM IMS-MS multi-pass platform; (**B**) To study different orientations, a mixture of PC(16:1(9Z):16:1(9Z)) and PC(16:1(9E):16:1(9E)) (*m*/*z* 730.539 for the protonated species) was studied again with one (15.9 m) and two passes (30.6 m). In (**A**) for the PC(18:1/18:1) lipids, the 6Z double bonds are shown in yellow and the 9Z are shown in blue; In (**B**) for the PC(16:1/16:1) lipids, the 9Z double bonds are shown in green and the 9E double bonds are shown in orange.

To initially explore structurally similar lipid isomers, we evaluated a series of glycerophosphatidylcholines (PCs), which comprise a large portion of cellular membranes and can have both saturated and unsaturated fatty acid chains. While the *cis* double bond configuration of the fatty acids is prevalent in eukaryotic organisms, *trans* fatty acids can be introduced via bacterial metabolism, and *cis* PCs can be converted to *trans* PCs by reaction with free radical species [43]. The first pair of PC standards studied were PC(18:1(6Z):18:1(6Z)) and PC(18:1(9Z):18:1(9Z)), which differed in the location of their double bonds (6th or 9th carbon) (Figure 3A), while the second pair PC(16:1(9Z):16:1(9Z)) and PC(16:1(9E):16:1(9E)), had the same double bond positions but different orientations, *cis* versus *trans* or Z versus E (Figure 3B). Figure 3 illustrates the utility of the multi-pass separation for distinguishing the two protonated isomer pairs with one (15.9 m) and two passes (30.6 m) in the SLIM IMS-MS platform. Initially, all four standards were run individually to determine their arrival times, and then mixtures for both pairs were analyzed to see if each isomer could be distinguished. For the isomers with different double bond placements, each was easily distinguished in the one- and two-pass analyses with the isomer having the double bonds at the 9th carbon having a larger structure than the isomer with the double bonds closer to the head group at the 6th carbon.

The *cis* versus *trans* pair presented more challenging but the isomer with *cis* double bonds was slightly more compact than the *trans* isomer. Thus, after one cycle, only partial separation was observed; however, an additional cycle allowed the peaks from the two components to be baseline resolved. While the *trans* double bonds only causes a minor change in structure from that of the totally saturated lipid, the *cis* double bonds significantly change the geometry, folding the fatty acid chain back inwards on itself and leading to a smaller three-dimensional structure. However, previous work using a conventional low pressure IMS drift tube platform only partially resolved these peaks for individual standards [2]. Thus, the baseline separation for both the double bond positions and orientations provides the basis for their evaluation in complex biological samples.

### 2.2. Glycolipid Isomers

**Figure 4:**
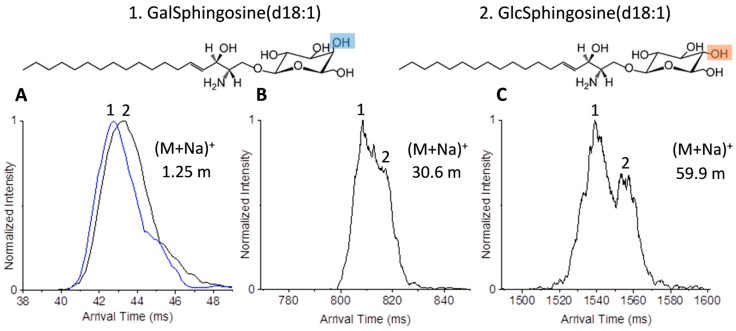
Ultra-high resolution IMS separations of sodiated GalSphingosine(d18:1) and GlcSphingosine(d18:1) (*m*/*z* 484.622). Separation of the individual isomers with (**A**) the 1.25 m path; and their mixture with (**B**) two (30.6 m) and (**C**) four passes (59.9 m). The hydroxyl group differences between the two isomers are highlighted in blue and orange.

Due to the separations achieved for the lipid isomers, we decided to explore the use of the multi-pass system with glycolipids which may have additional isomeric changes. Isomeric variations of glycolipids, in addition to those encountered in other lipid species, stem from changes in the glycan subunits identities, linkages, and branching which influence physico-chemical properties of the cellular membranes [44]. While glycolipids are categorized into three main groups (glycosylphosphatidylinositols, glycoglycerolipids, and glycosphingolipids (GSLs)), GSLs are prevalent in mammalian cell membranes and of great interest. In GSLs, the glycan moieties protrude beyond the membrane surface into aqueous media and mediate stereospecific recognition interactions to ions (for example Calcium exchange) [45], enzymes, antibodies and other molecules [44,46]. We first investigated galactosylsphingosine(d18:1) and glucosylsphingosine(d18:1) (denoted as GalSphingosine(d18:1) and GlcSphingosine(d18:1)), which are examples of the simplest glycolipids. Figure 4 shows the structural variation between the isomers, which stems only from the identity of the glycan (galactose or glucose) attached via a glycosidic linkage at the C1-hydroxyl moiety of the sphingosine hydrocarbon chain. These isomers are of great interest as they are the building blocks for higher-order glycosphingolipids and biomarkers of Gauchers and Krabbe disease [47,48]. In positive ion mode, GalSphingosine(d18:1) and GlcSphingosine(d18:1) readily form alkali metal adducts which may have affinity to different locations of the glycolipid molecules as indicated by their fragmentation patterns [49]. In our experiments, we investigated the (M + Na)^+^ form of each, which were the most abundant species observed. While hardly any separation was observed at 1.25 m for the individual isomers, partial separation of the sodiated species was achieved with one cycle (15.9 m) (Figure 4A) with GalSphingosine(d18:1) being a more compact structure than GlcSphingosine(d18:1). Upon analysis of the mixture, partial separation of the sodiated species was noted after four passes (59.9 m) (Figure 4C).

**Figure 5:**
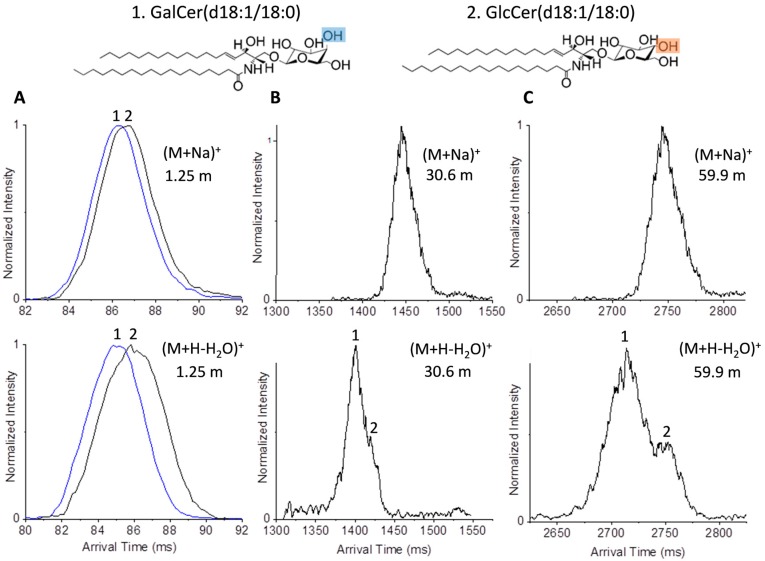
Ultra-high resolution IMS separations of the sodiated (*m*/*z* 750.604) and dehydrated (*m*/*z* 712.604) species of GalCer(d18:1/18:0) and GlcCer(d18:1/18:0). Separation of the individual isomers with (**A**) the 1.25 m path, and their mixture with (**B**) two (30.6 m) and (**C**) four passes (59.9 m). The hydroxyl group differences between the isomers are highlighted in blue and orange.

Next, we investigated GalCer(d18:1/18:0) and GlcCer(d18:1/18:0), which are more complex glycolipids that share the same length sphingosine hydrocarbon chain as GalSphingosine(d18:1) and GlcSphingosine(d18:1), but differ in the extra fatty acid attached to the sphingosine head group. When the standards for GalCer(d18:1/18:0) and GlcCer(d18:1/18:0) were studied in positive ion mode the prominent species for each was the labile protonated species which undergoes loss of water (M + H − H_2_O)^+^ [50]; however, the sodiated species for each was also observed. Upon analysis of the individual standards with the 1.25 m path, no useful separation was observed for the sodiated forms and only minor separation was observed for the dehydrated GalCer(d18:1/18:0) and GlcCer(d18:1/18:0) species (Figure 5A). Additional passes of the standards showed that separation could be obtained for the dehydrated forms but not the sodiated versions. Further, we observed that the dehydrated standards had broader peaks than the sodiated conformers. This could be due to the greater flexibility of the glycan head group in the absence of the sodium adduct and/or the products of dehydration at different positions [51]. Analysis of the mixture of GalCer(d18:1/18:0) and GlcCer(d18:1/18:0) showed that the sodiated form could not be resolved with either two or four passes. However, the dehydrated species showed partial separation in two cycles (Figure 5B) and further separation in four cycles (Figure 5C). These multi-pass analyses again showed that separation of GalCer(d18:1/18:0) and GlcCer(d18:1/18:0) were also possible with the multi-pass platform.

**Figure 6:**
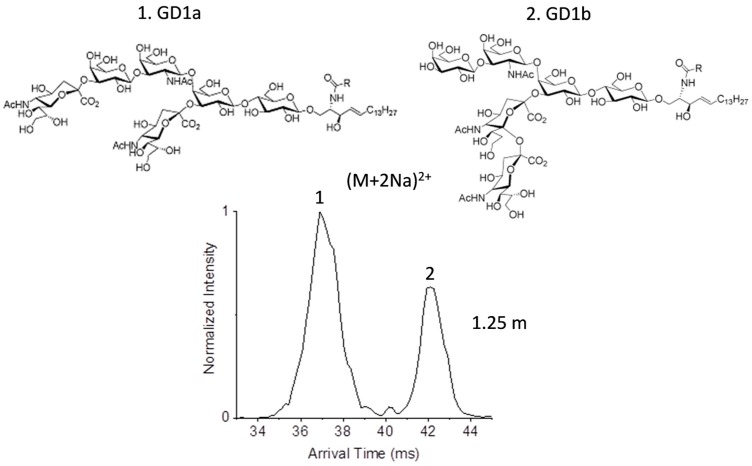
The ultra-high resolution IMS separation of the GD1a and GD1b ganglioside mixture for the (M + 2Na)^2+^ species of each isomer (*m*/*z* 941.476). This baseline separation enables each isomer to be easily distinguished in further assays.

As a final example, we examined gangliosides which are some of the most complex glycosphingolipids with glycan head groups containing one or more sialic acids. Gangliosides are present in mammalian brain and nervous system structures and have multiple functions including neural development differentiation and regulation of protein receptors. Two ganglioside isomers of great interest are the disialogangliosides GD1a and GD1b, which only differ in the localization of the sialic acid residues and whose antibodies are disease biomarkers in various neurological diseases [52,53]. In GD1a, one sialic acid residue is attached to the terminal galactose residue and another sialic acid residue is attached to the first galactose residue in the oligosaccharide head, while in GD1b the two sialic acids are attached to the first galactose residue in the oligosaccharide head. Gangliosides are typically analyzed with electrospray and tandem MS in positive or negative ion modes or with UV photodissociation [27,54,55]. Matrix-assisted laser desorption/ionization (MALDI)-IMS-MS studies previously achieved partial separation of each individual species of GD1a and GD1B, although failed to separate them in a mixture [56]. In our experiments, the most abundant species of GD1a and GD1b were (M + 2Na)^2+^. The doubled sodiated species of each isomer was analyzed individually and both illustrated a narrow peak and distinct drift times even with the 1.25 m path. When GD1a and GD1b were mixed, baseline resolution was observed for each isomer, indicating their ease of separation in more complex mixtures and potential to gain a better understanding of their functions (Figure 6).

## 3. Materials and Methods

### 3.1. Chemical Standards

All reagents were purchased from Sigma-Aldrich (St. Louis, MO, USA) in addition to the two disialoganglioside isomer standards (GD1a and GD1b). The glycerophosphatidylcholine (PC) standards (PC(18:1(9Z)/18:1(9Z)), PC(18:1(6Z):18:1(6Z)), PC(16:1(9Z)/16:1(9Z)), PC(16:1(9E)/16:1(9E)), PC(14:0/16:0), and PC(16:0/14:0)) and cerebroside standards (1-β-galactosyl-sphing-4-enine (psychosine or GalSpingosine), 1-β-glucosyl-sphing-4-enine (glucosylsphingosine or GlcSpingosine), C18 β-d-galactosyl ceramide (GalCer(d18:1/18:0)) and C18 β-d-glucosyl ceramide (GlcCer(d18:1/18:0))) were all purchased from Avanti Polar Lipids (Alabaster, Al). The isomers were each prepared at 5 µM concentrations in 95% methanol and 10 mM ammonium acetate and combined to form mixtures.

### 3.2. Instrumental Setup

Each lipid and glycolipid isomer was analyzed individually as well as in a mixture to determine the resolution of the isomer pairs. The solutions were electrosprayed using an infusion rate of 100–200 nL/min with a 20 µm inner diameter chemically etched emitter for SLIM IMS-MS analyses. The SLIM IMS-MS platform was similar to that of the 13 m serpentine design previously reported [57], but configured to allow multiple passes through the serpentine path for even higher IMS resolution (Figure 2). In this work, ions were accumulated in an ion funnel trap [58,59,60] and then released to the multi-pass SLIM device [61] which incorporated switches at the entrance and exit to the serpentine region, allowing selection of the number of passes. The minimal path length (switches disabled) was 1.25 m, and diversion into the 14.65 m long serpentine path allowed a total path length of 1.25 + 14.65*n* m, where *n* is the number of passes through the serpentine IMS region (Figure 2). The SLIM IMS was coupled to an Agilent 6224 Time-Of-Flight (TOF) MS with a 1.5 m extended flight tube via an ion funnel and RF quadrupole. All separations were performed at room temperature with the following parameters used: wave amplitudes of 30 V, guard electrode voltages of 15 V, and RF-confining potentials of 1.4 MHz frequency and 330 V peak to peak. Slight variations in traveling wave speed and pressure were applied in some cases: lipid separations were performed at 160 m/s travelling waves and ~2 Torr N_2_, while the glycolipid experiments used 90–100 m/s travelling waves and ~3 Torr N_2_ to further enhance the isomer resolution.

## 4. Conclusions

This manuscript provides an initial demonstration of a multi-pass Structures for Lossless Ion Manipulations (SLIM) ion mobility spectrometry-mass spectrometry (IMS-MS) platform achieving the ultra-high resolution necessary to distinguish lipids and glycolipids with very similar structures. Since the resolving power of ion mobility spectrometry (IMS) separations depends on the square root of the path length, the serpentine multi-pass platform offer a significant advantage for the analysis of structurally similar isomers. Further, distinguishing lipid and glycolipid isomers is essential for better understanding biological changes in complex environmental and biological samples and determining the potential roles and activities of these different isomers. The high peak capacity achievable with the multi-pass SLIM IMS-MS platform helps alleviate isotopic interferences associated with lower resolution IMS separations and enables many new applications. While the separations shown in this manuscript constitute a significant advancement in lipid and glycolipid IMS resolution, we note two remaining challenges for broader application. The first is the limited charge capacity (i.e., ion population size) that could be injected into the SLIM IMS-MS device, which ultimately limits peak detectability with increased number of passes; The second, and related, issue is the limited number of passes possible due to increasing peak widths, which reduce not only detection but also the range of mobilities that can be studied if “peak lapping” is to be avoided. In this regard, we note that our recent development of new IMS peak “compression” capabilities with SLIM enable greatly increased ion accumulation in the device itself, and also allows reduction of peak widths without any significant loss of resolution [62,63,64]. Thus, we anticipate more sensitive ultra-high resolution IMS separations in the future for structurally similar molecules, and the ability to do so in the context of “real-world” applications.
